# Recent progress on molecular breeding of rice in China

**DOI:** 10.1007/s00299-013-1551-x

**Published:** 2014-01-19

**Authors:** Yuchun Rao, Yuanyuan Li, Qian Qian

**Affiliations:** 1 State Key Laboratory of Rice Biology, China National Rice Research Institute, Hangzhou, 310006 China; 2 College of Chemistry and Life Sciences, Zhejiang Normal University, Jinhua, 321004 China

**Keywords:** Molecular breeding, Marker-assisted selection, Breeding design, Agronomic traits, Rice breeding

## Abstract

Molecular breeding of rice for high yield, superior grain quality, and strong environmental adaptability is crucial for feeding the world’s rapidly growing population. The increasingly cloned quantitative trait loci and genes, genome variations, and haplotype blocks related to agronomically important traits in rice have provided a solid foundation for direct selection and molecular breeding, and a number of genes have been successfully introgressed into mega varieties of rice. Here we summarize China’s great achievements in molecular breeding of rice in the following five traits: high yield, biotic stress resistance, abiotic stress resistance, quality and physiology. Further, the prospect of rice breeding by molecular design is discussed.

## Introduction

Rice is one of the most important staple crops in the world and serves as a model for monocots. In rice breeding, two breakthroughs have been made in China over the last century. The first breakthrough is the development of a semi-dwarf rice variety in the 1960s, which raised rice yield by more than 20 % per unit area; the second breakthrough is the development of a hybrid rice variety with the three-line or cytoplasmic male sterile system in the 1970s, which led to another great increase in average rice yield by 20 % (Yuan 1987). The previous achievements have contributed greatly to the self-sufficiency in China’s food supply. Thereafter, however, no substantial progress has been made in improving rice yield. In 1986 and 1996, International Rice Research Institute and China launched the Super Rice Breeding Program to fight in stages for increasing rice yield. Recently, rapid economic development and population growth have placed heavy pressure on crop production in China. To meet the security of food supply, we must increase the crop yield per unit area by 50 % before 2030 (Cheng and Hu 2008). In this context, it is imperative to find new applicable methods for rice breeding.

**Fig. 1:**
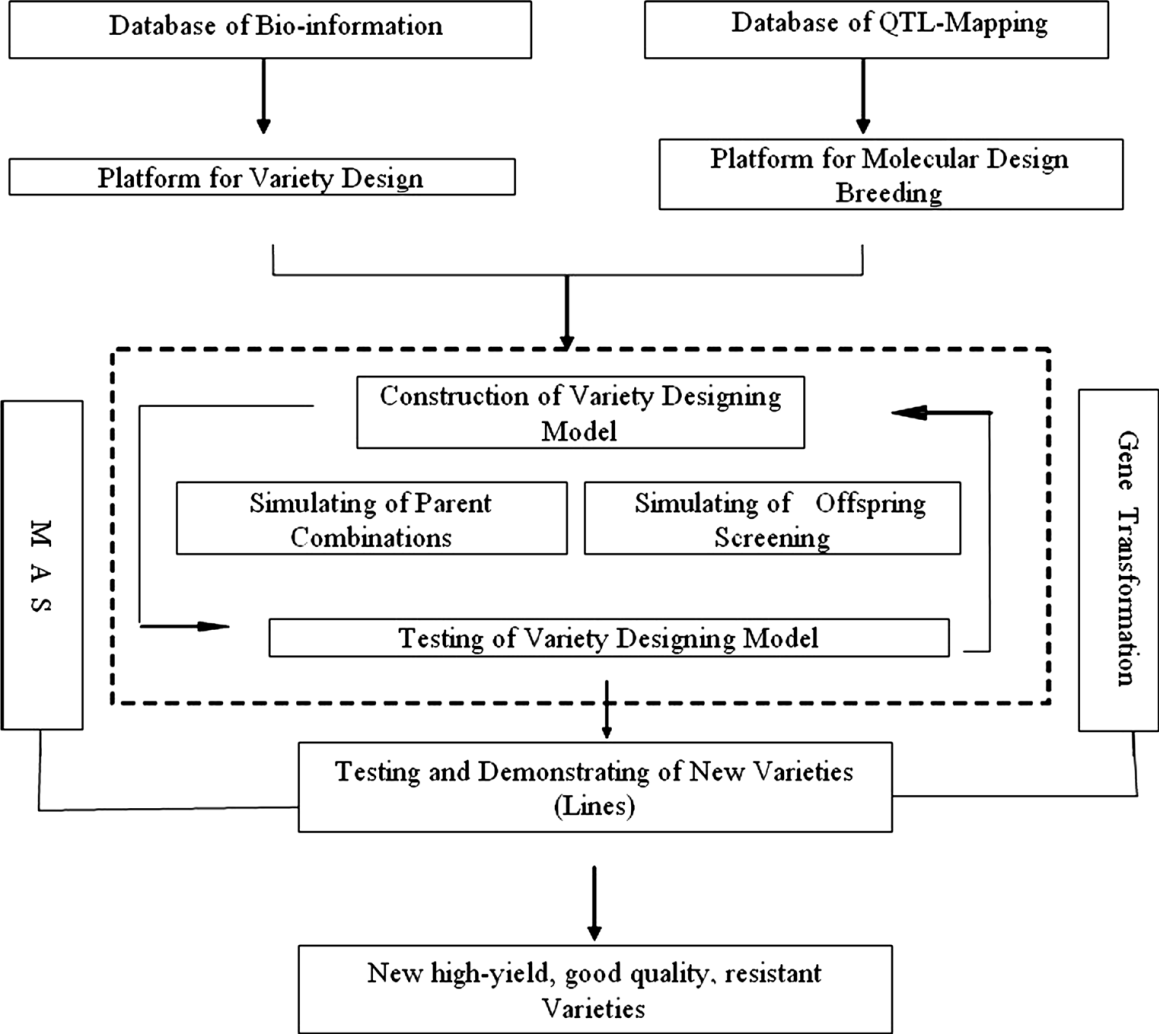
The scheme of applying MDB to breed new varieties

Conventional breeding selects genotypes indirectly through phenotypes, which is generally effective for qualitative traits only but not for quantitative traits. It is due to that quantitative traits with continuous variations are controlled by multiple genes and environmental factors. Over the past few decades, advances of molecular markers, transgenic technology, and genomics have exerted far-reaching influences on the concept and means of conventional rice breeding, allowing applications of molecular breeding technology in rice. Molecular breeding refers to the development of new rice varieties by integrating the means of modern biotechnology into conventional breeding methods (Fig. 1), which mainly involve marker-assisted selection (MAS) and genetic engineering breeding (GEB).

Compensating the deficiencies of conventional breeding, molecular markers designed for direct genotypic identification are unrestricted to the ontogenetic periods of plants and can be used to select target traits directly. MAS involves four steps: overall program design, selection of target genes and parental materials, construction of breeding populations, and molecular marker screening of early generation materials. The breeding process of MAS is similar to that of conventional breeding, except that in the former method, molecular marker detection is involved in every breeding generation on the basis of conventional phenotypic identification.

GEB of rice mainly involves in vitro recombination of a specific target gene with the transformation vector, followed by transferring into rice for stable integration, expression, and heredity. This breeding method avoids the impacts of adverse genes caused by genetic linkage in the process of sexual hybridization and gets rid of reproductive isolation between different rice cultivars. Therefore, GEB provides an efficient way for cultivating new rice varieties.

In recent years, great efforts have been made in rice genome sequencing and there have been significant developments of functional genomics. The increasingly cloned quantitative trait loci (QTL)/genes, genome variations, and haplotype blocks related to agronomically important traits in rice provide a solid foundation for direct selection and molecular breeding of rice. A number of genes are successfully transferred into mega rice varieties. Multiple chromosome segment substitution lines are constructed, and a large number of QTLs are identified. Many breeds carrying objective QTLs or genes are applied in rice production (Table 1), and an increasing number of rice varieties and genetic populations are sequenced, laying a foundation for rice breeding by molecular design. Here we summarize China’s current situation of molecular breeding in rice regarding different traits, and further discuss the prospects of rice breeding by molecular design (MDB).

**Table 1:** Examples of marker-assisted selection (MAS) and genetic engineering breeding (GEB) in rice

Varieties/lines	Types	Genes involved	Donors	Tolerance to/exploited traits	Breeding methods	Code/reference
Zhonghui 8006	*Indica*	*Xa21*, *GM6*	Duoxi 1, Minghui 63	BB, GM	MAS	CNA20030473.9
Zhonghui 218	*Indica*	*Xa21*	IRBB 21	BB	MAS	CNA20030093.8
Guodao 1	*Indica*	*Xa21*	Zhonghui 8006	BB	MAS	CNA20050721.4
Guodao 3	*Indica*	*Xa21*	Zhonghui 8007	BB	MAS	Cao et al. (2006)
Guodao 6	*Indica*	*Xa21*	Zhonghui 8008	BB	MAS	CNA20050722.2
IIYou 8006	*Indica*	*Xa21*	Zhonghui 8006	BB	MAS	Wu et al. (2008c)
IIYou-218	*Indica*	*Xa21*	Zhonghui 218	BB	MAS	CNA20060721.9
Yuanhui 611	*Indica*	*yld1.1*, *yld2.1*	*O.* *rufipogon*	High yield	MAS	CNA20030432.1
Y-You 7	*Indica*	*yld1.1*, *yld2.1*	Yuanhui 611	High yield	MAS	Wu et al. (2010a)
RB207-1	*Restorer*	*Barnyardgrass Genomic DNA*		High yield	GEB	CNA20030177.2
Shuhui 527	*Restorer*	*Xa4*, *Xa21*	1318/88-R3360	BB	MAS	CNA20000073.X
Zhunliangyou 527	*Indica*	*Xa4*, *Xa21*	Shuhui 527	BB	MAS	CNA20030033.4
D-You 527	*Indica*	*Xa4*, *Xa21*	Shuhui 527	BB	MAS	CNA20010111.0
Xieyou 527	*Indica*	*Xa4*, *Xa21*	Shuhui 527	BB	MAS	CNA20030434.8
RGD-7S/RGD-8S	*CMS*	*Pi1*, *Pi2*	BL122	RB	MAS	Liu et al. (2008b); Jin et al. (2007)
Yueza 746/763	*Indica*	*Pi1*, *Pi2*	RGD-7S	RB	MAS	
W3660	*Japonica*	*Lgc*-*1*	LGC-1	Low glutelin content	MAS	CNA20020113.1
W017	*Japonica*	*Lox3*	DawDam	Prolonged storage of seeds	MAS	CNA20020290.1
W025	*Japonica*	*ge*	Haiminori	Huge embryo	MAS	CNA20030548.4
Huahui 1	*Restorer*	*Cry1Ac/Cry1Ab*	Minghui 63	Insects	GEB	Liu et al. (2012)
Zhonghui 161	*Restorer*	*Pita*, *xa13*, *wx*	IRBB 51, Teqing	RB, BB, good quality	MAS	CNA20060673.5
Bph68S/Luohong4A	*CMS*	*Bph14*, *Bph15*	B5	BPH	MAS	Zhu et al. (2013)
Ning 9108	*Indica*	*Stv*-*bi*, *Wx*-*mq*	Guandong 194	Strip blight, good quality	MAS	Yao et al. (2010)
T16S	*GMS*	*Bt*	Minghui 63	Insects	GEB	Wu et al. (2010b)

BB, bacterial blight; RB, rice blast; GM, gall midge; BPH, blight planthopper; Bt, *Bacillus thuringiensis*; MAS, marker-assisted selection breeding; GEB, genetic engineering breeding

## Molecular breeding for high yield

High yield is the eternal theme pursued by rice breeders. Super rice breeding in the model of ideal plant architecture using molecular design is the mainstream of future development in this field. Yield-related traits in rice include plant height, tiller number, grain weight, and panicle type. Of these, plant height is the most important trait related to plant architecture and linearly correlates with biomass. Panicle number, which consists of planting density and effective tiller number, is a major influencing factor of the total grain production per unit area. Panicle characters are directly linked with the yield of rice (Xing and Zhang 2010). Recently, multiple yield-related genes and QTLs have been identified and cloned in rice (Table 2), providing a good opportunity for molecular breeding with greater potential of rice yield.

**Table 2:** Map-based cloning of genes using mutants or QTL as a tool involved in rice high yield in recent years

Trait	QTL or mutant	QTL/gene	RAP-DB	Encoded product	Reference
Plant architecture	mutant	*HTD2*	Os03g0203200	Putative esterase	Liu et al. (2009)
	mutant	*OsCD1*	Os12g0555600	Cellulose synthase	Luan et al. (2011)
	mutant	*OsGA2ox6*	Os04g0522500	Gibberellin 2-oxidase	Huang et al. (2010)
	QTL	*IPA1/WFP*	Os08g0509600	Squamosa promoter binding protein-like 14	Jiao et al. (2010)
	QTL	*OsPH1*	Os01g0881500	Chitin-inducible gibberellin-responsive protein	Kovi et al. (2011)
	mutant	*LAZY1*	Os11g0490600	Expressed protein	Li et al. (2007)
	mutant	*PROG1*	Os07g0153600	Cys2-His2 zinc finger protein	Jin et al. (2008)
	mutant	*DLT*	Os06g0127800	GRAS family protein (involved in brassinosteroid)	Tong et al. (2012)
Panicle characters	mutant	*EUI1*	Os05g0482400	Cytochrome P450 monooxygenase	Zhang et al. (2008)
	mutant	*sui1*	Os01g0118300	Phosphatidyl serine synthase	Zhu et al. (2011)
	mutant	*SP1*	Os11g0235200	Peptide transporter	Li et al. (2009b)
	mutant	*LAX2*	Os04g0396500	Nuclear protein with a plant-specific conserved domain	Hiroaki et al. (2011)
	QTL	*Ghd7*	Os07g0261200	CCT -domain protein	Xue et al. (2008)
	mutant	*DEP2/SRS1*	Os07g0616000	Novel plant-specific protein	Li et al. (2010a)
	mutant	*LAZY1*	Os11g0490600	Specific herb protein	Chen et al. (2012)
	mutant	*EG1*	Os01g0900400	Lipase	Li et al. (2009a)
	QTL	*DTH8/Ghd8*	Os08g0174500	OsHAP3 subunit of a CCAAT-box-binding protein	Wei et al. (2010)
	QTL	*DEP1/qPE9*-*1*	Os09g0441900	PEBP-like domain protein	Huang et al. (2009)
	mutant	*DEP3*	Os06g0677000	Patatin-like phospholipase A2 protein	Qiao et al. (2011)
	mutant	*OsPIN2*	Os06g0660200	Auxin efflux transporter	Chen et al. (2012)
Grain	QTL	*GS3*	Os03g0407400	Transmembrane protein	Yang et al. (2010)
	QTL	*Gn1a*	Os01g0197700	Cytokinin oxidase/dehydrogenase	Li et al. (2013)
	QTL	*GS5*	Os05g0158500	Serine carboxypeptidase	Li et al. (2011b)
	QTL	*GW2*	Os02g0244100	RING-type E3 ubiquitin ligase	Song et al. (2007)
	QTL	*GW5*	Os05g0187500	Novel nuclear protein	Weng et al. (2008)
	QTL	*GW8*	Os08g0531600	Squamosa promoter binding protein-like 16	Wang et al. (2012b)
Tiller	mutant	*MOC1*	Os06g0610350	GRAS family nuclear protein	Li et al. (2003)
	QTL	*TAC1*	Os09g0529300	Unknown	Jiang et al. (2012)
Heading and grain weight	mutant	*HGW*	Os06g0160400	Ubiquitin-associated domain protein	Li et al. (2012b)
Grain filling	QTL	*GIF1*	Os04g0413500	Cell wall invertase	Wang et al. (2010a)
Shattering	QTL	*SHA1*	Os04g0670900	Plant-specific transcription factor	Lin et al. (2007)

In 1991, the high-stem gene *eui* was first transferred into the widely used sterile line Zhenshan 97A by MAS. In the following year, this gene was transferred into other sterile lines using the backcrossing method to overcome the issue of elongated internodes of sterile lines (Liang et al. 1992). Recently, the dense and erect panicle 1 (*DEP 1*) gene, closely related to plant type, has been cloned (Huang et al. 2009). Using an elite indica variety (curved panicle type Nanhui 602) as female parent and a NIL-*DEP1* DW135 as male parent, researchers carried out MAS of backcross population, screened out a *DEP1*-containing homozygous line, and further investigated panicle traits in heading date; the results were consistent with molecular marker detection data, providing a theoretical reference and materials for future plant architecture breeding (Cheng et al. 2011).

Yang et al. (2010) and Wang et al. (2012b) effectively improved the grain size and the exterior quality of an indica variety, Huajingxian 74, by molecular pyramiding breeding, which involved the hybridization of a single segment substitution line that has genetic background of Huajingxian 74 and carries the grain length genes *GS3* and *GW8* with other excellent genes. Recently, the characteristics of panicle size have been improved significantly by clustering 8 panicle number and weight-related QTLs via MAS (Zong et al. 2012). Meanwhile, the strong restorer line Q611 and the hybrid rice variety Y-You-7 were obtained by transferring two high-yield genes of the Malaysian wild rice *Oryza* *rufipogon*, *yld1.1* and *yld2.1* (Wu et al. 2010a), into the elite restorer lines Ce64-7 and 9311 via MAS (Table 1).

In 2003, the State Key Laboratory of Rice Biology (SKLRB) of China National Rice Research Institute (CNRRI) cooperated with the research group of Academician Li Jiayang from Chinese Academy of Sciences to complete the cloning of rice *monoculm 1* (*MOC1*) gene (Li et al. 2003). With the help of *MOC1* cloning, the SKLRB of CNRRI launched a research project funded by the 863 program, *Creation and Applications of Super High*-*yielding Germplasm of MOC1 Transgenic Rice*. This project mainly aimed to transfer sense, antisense, and deletion genes of *MOC1* into mega variety and the parents of hybrid rice for changing *MOC1* expression level and regulating rice tiller numbers.

In addition, GEB has been applied for screening transgenic plants with less or no tillers, good agronomic traits, and great potential for high yield. To date, more than 50 transgenic lines have been bred using the pedigree methods and 30 transgenic intermediate materials are obtained. Of these, three transgenic varieties were further selected for significantly improved production and potential for application compared to the control lines; four lines with fewer tillers were used for variety demonstration; and some lines were used as intermediate materials. In addition, *MOC1* transgenic pure lines were hybridized with conventional varieties such as Zhi-7 and Zhongchao-123, and a series of *MOC1*-containing breeding materials from different generations were obtained by transformation, including 40 intermediate materials from advanced lines.

Previous work indicates that using modern genetic engineering technology, we are able to obtain transgenic plants with fewer tillers, high nutrient contents, and superior agronomic traits in super rice breeding. The gradient tiller materials generated by transgenosis are expected to provide a new platform for exploring the theory and technology of super rice breeding. With the cloning of *IPA1*, great breakthroughs have been made in understanding the mechanism of ideal plant architecture in rice. It has been reported that *IPA1* contributes mostly to more panicles, strong culms, and high-yield potential. This gene has been transferred into the rice cultivar Xiushui 11 through backcross breeding and the obtained mutant lines exhibit ideal plant architecture with a 10 % increase in the yield in field experiments (Jiao et al. 2010).

In overseas, great achievements also had been acquired. Previously, the plant height gene *sd1* was transferred from the variety Habataki into the variety Koshihikari using MAS; near-isogenic lines NIL-*GN1* + *sd1*, a kind of semi-dwarf, large-panicle, high-yielding lines, were constructed (Ashikari et al. 2005), providing a new pathway for the green revolution in rice.

## Molecular breeding for resistance to biotic stress

In rice production, biotic stress mainly refers to plant diseases and pests. The major rice diseases are fungal, bacterial, and viral diseases and rice blast. Serious losses to rice production are commonly caused by more than 70 diseases, of which rice blast, sheath blight, and bacterial blight are most harmful to rice. In addition, rice is one of the crops suffering from most pests’ attacks. There are more than 624 insect species in field harmful to rice, of which planthopper, leafhopper, and stemborer cause the most serious hazards and lead up to 32 % yield losses in rice (Pei et al. 2011). Controlling of the pests and breeding of disease-resistant varieties have long been the focus of rice research. In China, a number of exploratory studies have been conducted on plant disease resistance in rice, and a series of relevant genes (e.g., *Xa21*) have been cloned and applied in rice production (Table 3). These works have greatly promoted rice breeding for high resistance to biotic stress by MAS and GEB.

**Table 3:** Major important genes tagged and mapped with molecular markers in rice for biotic stresses in recent years

Biotic stress	*Gene*	Donor	Chr.	Linked marker	Reference
Bacterial blight	*Xa13*	IRBB13	8	R2027 (1.3 cM), RG136(2.3 cM)	Li et al. (2012a)
	*Xa4*	IRBB4	11	R1506, s12886 (0.5 cM)	Deng et al. (2005)
	*Xa7*	IRBB7	6	G1091 (6.0 cM), AFLP31-10 (3 cM)	Porter et al. (2003)
	*Xa21*	*O. longistaminata*	11	RG103 (0 cM)	Gan et al. (2011)
	*Xa22(t)*	Zhachanglong	11	RG103 (0 cM)	Pei et al. (2011)
	*Xa23*	*O.* *rufipogon*	11	C1003A (0.4 cM)	Chen et al. (2009)
	*xa24*	DV85, DV8, Aus 295	2	RM14222 (0.07 cM), RM14226 (0.07 cM)	Wu et al. (2008d)
	*Xa25(t)*	Minghui63	12	G1314 (7.3 cM), R887	Pei et al. (2011)
	*Xa29*	*O.* *officinalis*	1	C904, R596	Tan et al. (2004)
	*xa32(t)*	Y76	12	RM8216 (6.9 cM)–RM20A (1.7 cM)	Ruan et al. (2008)
	*xa34(t)*	BG1222	1	RM10929, BGID25	Chen et al. (2011b)
Rice blast	*Pi1*	LAC23	11	RZ536 (7.9 cM), Npb181 (3.5 cM)	Hua et al. (2012)
	*Pi2/Pi9*	5173	6	RG64 (0.9 cM), AP22 (1.2 cM)	Zhu et al. (2012)
	*Pi9(t)*	*Oryza minuta*	6	Pb9-1	Chen et al. (2009)
	*Pid3/Pi25*	Gumei2	6	A7 (1.7 cM), RG456 (1.5 cM)	Chen et al. (2011a)
	*Pi33*	IR64	8	Y2643L (0.9 cM), RM72 (0.7 cM)	Miah et al. (2013)
	*Pik*-*p*	K60	11	RM5926-K37	Yuan et al. (2011)
	*Pi41*	Nov-93	12	STS40-1–STS40-3	Yang et al. (2009b)
	*Pid(t)*	Digu	2	G1314A (1.2 cM), G45 (10.6 cM)	Pei et al. (2011)
Brown planthopper	*Bph6*	*Swarnalata*	11	RM6997–RM5742	Qiu et al. (2010)
	*Bph14*	*O. officinalis*	3	G1318–R1925	Zhu et al. (2013)
	*Bph19(t)*	AS20- 1	3	RM6308–RM3134	Chen et al. (2006)
White-backed planthopper	*Wbph6(t)*	Guiyigu	11	RM167–RM287	Li et al. (2010a, b)
Gall midge	*GM6*	Duokang1	4	PSM101, PSM106, PSM115	Xiao et al. (2005)
Stripe disease	*stv*-*bi*		11	ST10	Yao et al. (2010)
Insects	*Bt*	*Bacillus thuringiensis*			Chen et al. (2009)

Bacterial blight and rice blast resistance genes are most commonly used in rice breeding for disease resistance. Since the bacterial blight-resistant gene *Xa21* is tightly linked to the molecular marker PTA248, researchers from CNRRI detected homozygous-resistant plants using the molecular marker in the offspring of a cross of *Xa21*-containing variety IRBB21 and non-*Xa21*-containing variety IR24; two *Xa21*-carrying restorer lines, Zhonghui 8006 and Zhonghui 218 (Table 1), were bred, and a series of super rice combinations were obtained, such as Guodao 1 (Cao et al. 2005), Guodao 3 (Cao et al. 2006), Guodao 6 (Wu et al. 2008b), and II You 8006 (Wu et al. 2008c). In addition, maintainer lines pyramiding three rice blast resistance genes (*Pi*-*1*, *Pi*-*2*, and *Pi*-*33*) and maintainer and restorer lines pyramiding a rice blast resistance gene (*Pi*-*25*) with two bacterial blight resistance genes (*Xa*-*23* and *Xa*-*21*) were created. With the help of MAS, new resistance rice lines pyramiding multiple bacterial blight resistance genes (*Xa4*, *Xa13*, and *Xa21*) were screened out from the offspring of a cross between the variety IBRR60 and multiple disease-resistant varieties (Deng et al. 2005). In recent years, these rice varieties have been applied in rice production in an average annual area of approximately 35,000 hm^2^, which are considered to be successful examples for commercial application of bacterial blight resistance genes.

Using MAS, Wang et al. (2004b) transferred two bacterial blight resistance genes, *Xa21* and *Xa4*, into restorer lines, bred the disease-resistant, high-affinity restorer line Shuhui 527, and configured a combination of two-line hybrid rice Zhunliangyou 527 and three-line hybrid rice D You 527, Gangyou 527, and Xieyou 527 (Table 1). In addition, Liu et al. (2008b) and Jin et al. (2007) transferred *Xa7* into three-line restorer lines and bred the restorer lines Guanghui 806 and Guanghui 312. *Pi*-*1* and *Pi*-*2* were also, respectively, transferred into the sterile lines GD-7S and GD-8S for breeding the new sterile lines RGD-7S and RGD-8S (Table 1) for high resistance to rice blast, and new combinations of two-line hybrid rice with high resistance to rice blast were screened out, including Yueza 746, Yueza 751, Yueza 4206, and Yueza 750.

On the other hand, gene loci were detected in segregating generations of Wuyunjing 8 and Zhendao 42 using gene markers of *Pi*-*ta* and *Pi*-*b* and molecular markers tightly linked with *Stv*-*bi*; the three disease resistance genes were transferred simultaneously into high-yield varieties to breed a high-yield, good-quality, and multi-resistance new rice line, 74121, by a combination of multi-generation breeding in field with resistant gene identification (Wang et al. 2011). In another study, the rice blast resistance gene *Pi*-*1* was transferred into the three-line sterile line Jinkang A (Guan et al. 2009). Recently, continuous breakthroughs have been made in molecular breeding of rice for resistance to stripe diseases. Pyramiding breeding was carried out by configuring hybrid combinations between the Jiangsu high-yielding rice variety Wuyunjing 7 as female parent and the Japanese japonica variety Guandong 194 (containing the stripe disease resistance gene *Stv*-*bi* and dark endosperm mutant gene *Wx*-*mq*) as male parent to breed new disease-resistant rice lines with good cooking quality (Yao et al. 2010).

In rice molecular breeding for high resistance to pest, the elite two-line restorer line Yangdao 6 was hybridized with a selectable marker-eliminated *Bt* transgenic restorer line Minghui 63 to breed the transgenic pest-resistant photo-thermo-sensitive genic male sterile line T16S (Wu et al. 2010b). In 2013, the Honglian-type new sterile line Luohong 4A resistant to brown planthopper (BPH) was bred in Wuhan University by a combination of MAS and conventional breeding; Luohong 4A demonstrated significant BPH resistance by pyramiding two BPH-resistant genes, *Bph14* and *Bph15* (Zhu et al. 2013).

Xiao et al. (2005) transferred the rice gall midge (RGM)-resistant gene *Gm6* into the restorer lines Gui99 and Guanghui 998 using the gene marker PSM101 and preliminarily bred the RGM-resistant restorer lines KG18-1 and KG18-2. In September 2009, the Ministry of Agriculture issued a security certificate to the *cry1Ab/cry1Ac* transgenic insect-resistant rice Huahui 1 and *Bt* Shanyou 63 for production permissions in Hubei Province (Liu et al. 2012). Scientists from Science Academy of China created new-type pest-resistant transgenic materials and carried out bio-safety evaluations for non-selectable marker *sck/cry1Ac* double gene insect-resistant transgenic rice lines, derived varieties, and their hybrid combinations. Zhang et al. (2013b) created three copies of new restorer materials containing the major QTLs, *qSI4*, of anti-feeding resistance to white-backed planthopper by a combination of MAS and conventional breeding.

For cultivating new rice varieties resistant to both plant diseases and pests, multi-gene pyramiding breeding has been carried out. For example, genes highly resistant to bacterial blight (*Xa23*), rice blast (*Pi9*), and rice stem borer and leaf roller (*Bt*) were pyramided into the same lines using MAS to obtain a pure line with comparable resistance to specific biotic stresses mentioned above (Chen et al. 2009). In addition, two anti-aging genes (*IPT* and *Xa23*) and a rice blast resistance gene (*Pib*) were pyramided into the same lines to obtain anti-aging, bacterial blight-resistant, and rice blast-resistant intermediate materials for crossbreeding (He et al. 2004).

## Molecular breeding for resistance to abiotic stress

Abiotic stress of rice is collectively referred to natural adversity and artificial adversity. Natural adversity includes meteorological disasters (e.g., floods, droughts, typhoons, and cold damage) and soil adversity (e.g., salt damage); artificial adversity is mainly human-induced environmental pollution. In recent years, molecular breeding for abiotic stress tolerance in rice has been developed significantly, and a series of genes and QTLs have been identified for their high application potential in rice breeding for resistance to adversity (Table 4).

**Table 4:** Major important genes tagged and mapped with molecular markers in rice for abiotic stress, quality, physiology and hybrid traits in recent years

Trait	Gene	RAP-DB	Encoded product	Gene function	Reference
Abiotic resistance	*OsTPP1*	Os02g0661100	Trehalose-6-phosphate phosphatase	Tolerance to salt and cold	Ge et al. (2008)
	*OCPI1*	Os01g0615100	Chymotrypsin inhibitor	Tolerance to drought	Huang et al. (2007)
	*LTN1*	Os05g0557700	Ubiquitin–conjugating domain protein	Signal of Pi starvation	Hu et al. (2011)
	*OsLEA3*-*1*	Os05g0542500	Late embryogenesis abundant protein	Tolerance to drought	Xiao et al. (2007)
	*OsPht1*	Os10g0444700	Phosphate transporter	Absorption and transport of Pi	Jia et al. (2011)
	*OsSKIPa*	Os02g0759800	Unknown	Tolerance to drought	Hou et al. (2009)
	*LOX3*	Os03g0700400	Lipoxygenase	Tolerance to various stress	Liu et al. (2008a)
	*OsCOIN*	Os01g0104100	Zinc finger protein	Tolerance to salt, cold and drought	Liu et al. (2007b)
	*OsHAL3*	Os06g0199500	Halotolerance protein	Tolerance to salt	Sun et al. (2009)
	*SKC1*	Os01g0307500	Na^+^-selective transporter	Tolerance to salt	Ren et al. (2005)
	*SNAC2*	Os01g0884300	Nuclear protein	Tolerance to salt and cold	Hu et al. (2008)
Quality	*OsVPE1*	Os04g0537900	Vacuolar processing enzyme	Maturity of gluten	Wang et al. (2009b)
	*RSR1*	Os05g0121600	Unknown	Regulating starch synthesis of seeds	Fu and Xue (2010)
	*OsRab5a*	Os12g0631100	Small GTPase	Transporting storage protein	Wang et al. (2010b)
	*ALK*	Os06g0229800	Soluble starch synthases	Regulating gel temperature	Gao et al. (2003)
	*qGC*-*6(wx)*	Os06g0133000	Granule-bound starch synthase	Regulating amylase content	Su et al. (2011)
	*OsBADH2/fgr*	Os08g0424500	Betaine aldehyde dehydrogenase	Fragrance	Chen et al. (2008)
Physiology	*Phr1*	Os04g0624500	Polyphenol oxidase	Browning seeds	Yu et al. (2008)
	*OsMST6*	Os07g0559700	Monosaccharide transporter	Regulating grain filling	Wang et al. (2008)
	*SLL1*	Os09g0395300	KANADI Transcription Factor	Regulating leaf morphology	Zhang et al. (2009)
	*LC2*	Os02g0152500	Unknown	Regulating leaf angle and flowering	Wang et al. (2013)
	*Roc5*	Os02g0674800	Leu-chain-like protein	Regulating leaf morphology	Zou et al. (2011)
	*NLS1*	Os11g0249000	Typical CC-NB-LRR domain protein	Resistance to pathogens	Tang et al. (2011)
	*SL1*	Os01g0129200	Z-finger domain protein	Regulating rice floral development	Xiao et al. (2009)
	*NRL1/OsCSLD4*	Os12g0555600	Cellulose synthase	Cell-wall synthesis and plant growth	Yoshikawa et al. (2013)
Physiology	*OsRAA1*	Os01g0257300	12 kDa small G protein	Regulating root growth	Han et al. (2008)
	*WOX11*	Os07g0684900	Unknown	Regulating root growth	Zhao et al. (2009)
	*OsC6*	Os11g0582500	Lipid transfer protein	Involving in anthers development	Zhang et al. (2010b)
	*OsPSS1*	Os04g0573000	Pi-transport protein	Involving in Pi balance of leaves	Wang et al. (2012a)
	*PTC1*	Os09g0449000	PHD-Finger Protein	Involving in anthers development	Li et al. (2011a, b)
	*OsJAG*	Os01g0129200	C2H2 Z-finger domain protein	Involving in floral development	Duan et al. (2010)
	*CYP704B2*	Os03g0168600	Cytochrome P450	Regulating anther and pollen formation	Li et al. (2010b)
	*OsMST4*	Os03g0218400	Monosaccharide transporter	Regulating sugar distribution	Wang et al. (2007)
	*OsYABBY4*	Os02g0643200	YABBY-domain protein	Expressing in vascular tissues	Liu et al. (2007a)
	*SDG714*	Os01g0927000	Histone H3K9 methyl-transferase	Regulating leaf morphology	Ding et al. (2007a)
	*OsAGO7*	Os03g0449200	Argonaute (AGO) protein	Regulating leaf morphology	Shi et al. (2007)
	*RID1*	Os10g0419200	Cys2/His2 type z-finger transcription factor	Regulating reproductive growth	Wu et al. (2008a)
	*S5*	Os06g0213100	Aspartic protease	Regulating reproductive isolation	Ji et al. (2012)
	*Sa*	Os01g0578700	Small ubiquitin-like modifier E3 ligase-like protein/F-box protein	Regulating male sterility	Long et al. (2008)
	*EUI1*	Os05g0482400	Cytochrome P450 monooxygenase CYP714D1	Hybrid rice pollination	Zhang et al. (2008)
	*OsUgp1*	Os09g0553200	UDP-glucose pyrophosphorylase	Male fertility	Chen et al. (2007)
	*CSA*	Os01g0274800	R2R3-type MYB transcription factor	Mutation causing sensitive male sterility	Zhang et al. (2013a)
	*orfH79*	Mitochondria	Cytotoxic peptide	Regulating in HL-CMS	Peng et al. (2010)
	*COX11*	Os03g0718600	Nuclear-encoded mitochondrial protein	Interacting with WA352	Luo et al. (2013)
	*WA352*	Mitochondria	Unknown	Regulating in WA-CMS	
	*Rf5/Rf1*	Os10g0497300	PPR protein	Restoring fertility of BT-CMS	Hu et al. (2012)

Drought resistance is a complex trait in plants, which refers to the tolerance of plants to water-deficient environment. The existing technical evaluation and standards for drought resistance cannot reflect the actual growth conditions of plants accurately. In China, studies have been reported on QTL mapping of drought resistance traits in rice and a number of relevant genes cloned (Table 4). Eight QTL for rice root traits were identified in the doubled haploid (DH) lines Zhaiyeqing 4 and Jingxi 17 (Xu et al. 2001), and two drought-tolerant QTL, *qDT5* and *qDT12*, were detected at seedling stage and located at GA41–GA257 on chromosome 5 and RG457–Y12817R on chromosome 12 (Teng et al. 2002). Drought-tolerant QTLs including root traits (diameter, length, weight, and root/shoot ratio), leaf water potential and osmotic potential, as well as plant height, stem diameter, and flag leaf length and width were identified in DH lines of two japonica rice varieties, IRAT 109 and Yuefu, under different environmental conditions (root irrigation, potted paddy field, and dry land); in total, 21 additive QTL and 23 pairs of epistatic QTL were detected, and environmental interaction was detected in QTLs of root number, root fresh weight, root dry weight, and root/shoot ratio, but not in QTLs of basal root thickness, maximum root length, fresh stem weight, or dry stem weight; overall, the QTLs controlling fresh and dry root weights had the most significant interactions with the environment, accounting for 26 and 28 % of the variance, respectively (Mu et al. 2003). In addition, drought-related QTLs such as those associated with fresh root weight and leaf water potential were identified in different populations of recombination inbred lines (RILs) in paddy field and dry land (Qu et al. 2008). Knowledge of these QTLs will benefit MAS breeding of rice for drought resistance.

Cold damage of rice occurs at the budding, seedling, booting, flowering, and grain filling stages. Of these, anti-cold stress in the seedling stage is the focus of relevant research. QTLs related to cold stress have been identified with different methods (Table 4). Backcross generation was analyzed in 213 lines of Xieqingzao B and Dongxiang wild rice with the seedling mortality rate at low temperature as an indicator; it was found that the seedling mortality rate was continuously distributed in the population, that is, cold tolerance is a quantitative trait controlled by multiple genes; further, the major effect QTL was found on chromosome 8 in rice (Rao et al. 2013).

Wang et al. (2009c) investigated a set of RILs using the germination rate under low-temperature stress as an indicator and detected seven QTLs for cold tolerance on chromosomes 4, 6, and 9 in rice. The majority of known QTLs for cold tolerance are mainly located on chromosomes 4 and 8, accounting for 16.22 and 13.51 % of the total QTLs, respectively (Rao et al. 2013). At present, few studies have attempted to enhance cold tolerance of rice by molecular breeding. This is probably because cold tolerance in rice is a cumulative trait regulated by multiple genes, and single-gene transformation techniques have limited efficiency in improving rice cold tolerance.

Salt damage is one of the important causes for decline in rice yield and saline-alkali soil covers an area of approximately 100 million hm^2^ in China (Hu et al. 2010). To date, a few genes for salt tolerance have been cloned (Table 4) and breeding practices for salt resistance have been carried out using GEB. In 2006, the salt tolerance gene *OPBP1* was transferred into rice using a gene gun method and the obtained transgenic plants showed faster growth with significantly higher chlorophyll content and biomass yield than the non-transgenic control (Li and Guo 2006). The rice *HAL2*-like gene (*RHL*) was transferred into the japonica variety Hejiang 19 using an *Agrobacterium*-mediated method, and the screened positive plants showed improved salt tolerance at the seedling stage with less damage to cell membrane, strong vitality of leaf tissues, and enhanced salt tolerance under salt stress at the booting stage (Hu et al. 2010). The 2-pyrrol 5-carboxylate synthase (*P5CS*) gene from leguminous plants was transferred into rice using the gene gun method and the transgenic plants obtained increased content of proline with enhanced salt tolerance in transgenic cells (Zhi et al. 2005). In addition, transgenic rice plants with enhanced salt tolerance were obtained by transferring single genes such as 1-phosphate mannitol dehydrogenase (*mtlD*) gene and 6-phosphate, sorbitol dehydrogenase (*gutD*) gene, or double genes such as choline monooxygenase (*CMO*) gene/betaine aldehyde dehydrogenase (*BADH*) gene into rice varieties (Hu et al. 2010).

In 2006, Guo et al. (2006) transferred five salt tolerance-related genes, *CMO*, *BADH*, 1-phosphate mannitol dehydrogenase (*mtlD*) gene, *gutD*, and S-adenosylmethionine decarboxylase (*SAMDC*) gene, into the conventional *japonica* varieties Xiushui 11 and Zhonghua 11, *indica* varieties Teqing and hybrid restorer line Minghui 63 using *Agrobacterium*-mediated and gene gun methods; the five salt tolerance genes were then pyramided through conventional cross breeding and the rice line Xiushui 11 with nine genotypes was bred; further, these rice lines were chosen for comprehensive evaluation and effective utilization in south Zhejiang Province. Using map-based cloning, Ren et al. (2005) isolated the gene *SKC1* that is involved in regulating K(+)/Na(+) homeostasis under salt stress, providing a potential tool for improving salt tolerance in crops.

In China, few studies have investigated submergence stress in rice. New submergence-tolerant germplasm such as 94D-05, 94D-34, and 94D-54D have been obtained using an exogenous DNA introduction method, and the varieties Guizhao 2, Hui 41, 8105/D100, and Shanyou 63 with strong submergence tolerance were screened out under artificial simulation conditions (Li and Li 2000).

## Molecular breeding for rice grain quality

Rice quality, as jointly determined by the appearance, processing, cooking, eating, and nutrition of rice, is the characteristic of rice commercialization and industrialization. In China, many genes and QTLs related to rice quality have been cloned (Table 4), and some genes are used in practices of breeding.

Using MAS with the functional marker GRM04, new hybrid rice materials with strong fragrance were bred in Guangdong Academy of Agricultural Sciences by introducing fragrant (*fgr*) gene into the three-line maintainer lines Tianfeng B, Rongfeng B, Taifeng B, and Zhenfeng B as well as the elite restorer lines Guanghui 998, Guanghui 290, and Guanghui 372. New *wx*-containing rice lines with low content of amylose were developed (1.36 % in D154, 14.28 % in D156, and 13.13 % in D174 vs. 26.8 % in Tianfeng B).

Hybrid combinations were configured for pyramid breeding using Wuyunjing 7 as female parent and the *Stv*-*bi*- and *Wx*-*mq*-carrying Guandong 194 as male parent; the marker SCAR co-segregated with *Stv*-*bi* and the functional marker CAPS linked to *Wx*-*mq* were used to detect the target loci in segregating generations; *Stv*-*bi* and *Wx*-*mq* were simultaneously transferred into high-yielding rice varieties, and a new rice line (Ning 9108) with improved quality, disease resistance, yield, and agronomic traits was screened out and bred by a combination of field breeding, resistance identification, and grain endosperm appearance identification (Yao et al. 2010). Wang et al. (2009a) firstly designed InDel markers in the coding region of *ALK* and *fgr*, which controlled gelatinization temperature (GT) and dominated the trait of rice fragrance; these two genes were successfully introgressed in Minghui 63 lines according to the progress of backcross pyramiding using the two developed molecular markers; the improved Minghui 63 lines exhibited significant low GT and high gel consistency (GC), while the white core chalkiness decreased and fragrant trait expressed in these lines; the results elucidated that the quality of Minghui63 has been improved significantly in terms of rice appearance, cooking, and eating quality.

## Molecular breeding for improved physiological traits

High-photosynthetic efficiency is one of the most physiological traits for enhancing rice biomass and grain yield potential. In China, a number of rice genes related to physiological traits have been cloned (Table 4). These genes have been used in molecular breeding of rice for high-photosynthetic efficiency as a new means of physiological breeding, relative to rice breeding of plant architecture in the 1960s. The focus of rice breeding for high-photosynthetic efficiency is to explore potential of the single-leaf photosynthetic rate.

C4 plants are considered to have higher photosynthetic efficiency than C3 plants by lower photorespiratory consumption. Phosphoenolpyruvate carboxylase (PEPCase) is the key enzyme for CO_2_ fixation in C4 plants. A maize *PEPC* gene was transformed into rice variety to produce new transgenic rice materials. Scholars have investigated the possible mechanisms of unique CO_2_ assimilation and high-photosynthetic efficiency in the rice variety carrying *PEPC*; high-yielding rice plants with stable high-photosynthetic efficiency were obtained by system selection and multi-generation cultivation; finally, high-yielding rice varieties with high-photosynthetic efficiency were bred (He et al. 2005). The attempts of rice breeding for high-photosynthetic efficiency by a combination of conventional breeding and molecular biotechnology provide new approaches for breeding super rice in the future.

Physiological analysis demonstrated that PEPCase activity of Kitaake-*PEPC* transgenic rice varied in the tillering and heading stages, as well as different growth stages of flag leaf, all significantly higher than that in the parent Kitaake (He et al. 2005). Ding et al. (2007b) studied the photosynthetic characteristics of sorghum C4-type *PEPC* transgenic rice and found that CO_2_ compensation point and photorespiration rate significantly decreased while light-saturated photosynthetic rate and carboxylation rate increased in transgenic plants, reflecting the photosynthetic characteristics of C4 plants. Wang et al. (2004a) also indicated that the major economic traits of *PEPC* transgenic rice, including effective panicles per plant, total grains per panicle, thousand-grain weight, and yield per plant, were respectively improved by 14.9, 5.7, 1.3, and 13.9 % compared to those of the original parent Kitaake.

Regarding the same traits of *PEPC* + *PPDK* transgenic rice, the effective panicles and yield per plant were, respectively, improved by 29.1 and 27.0 % compared to the receptor parent Kitaake (He et al. 2005). In BC_1_F_1_, BC_2_F_1_, and BC_1_F_2_ of *PEPC*-containing Shuhui 881, the numbers of tillers, effective tillers, thousand-grain weight, and regenerated shoots all increased compared to those of the control variety Shuhui 881. The above studies have laid a foundation for molecular breeding of high-photosynthetic efficiency in super rice. In addition, researchers from the National Hybrid Rice Engineering Technology Research Center (Changsha City, Hunan Province) have mapped multiple high-yield, high-photosynthetic efficiency genes (Table 4), identified dozens of functional molecular markers and candidate genes, and created a large amount of germplasms with high-photosynthetic efficiency and the genes of key photosynthetic enzyme in C4 plants.

In China, rice heterosis is mainly utilized by cultivating male sterile line, male sterile maintainer line, and male sterile restorer line (collectively referred to as three-line), so as to select dominant combinations (Sun et al. 2012). In recent years, restorer genes and wide compatibility genes have been successively cloned along with rapid development of biotechnology (Table 4), and the heterosis in indica and japonica subspecies has been gradually shifted from exploratory research to practical application.

The wide compatibility gene *S5* of rice was cloned in 2008 (Yang et al. 2012) and rapidly used in production practices thereafter. By designing molecular markers of *S5*, Yang et al. (2009a) have screened out two restorer lines and a large amount of new rice germplasms carrying the wide compatibility gene *S5*-*n*. The functional markers of *S5*-*n* were useful to identify rice resources and detect hybrid purity (Zhang et al. 2010a).

## Prospects

In recent years, great progress has been made on molecular breeding in rice and the per unit area yield of this crop has been significantly improved in China. What then is the best path to increasing grain yield and further improve the quality of rice?

First is to pay more attention to the development and utilization of wild rice. Narrow genetic background of parent materials is the main cause for the undesirable crop yield, quality, and resistance, while wild rice provides rich genetic resources with good traits such as strong pest resistance and stress tolerance. Second is to understand the vital role of wide compatibility resources in rice heterosis. Wide compatibility rice materials have been continuously discovered and applied to hybrid rice breeding. Wide compatible genes can overcome the hybrid obstacles in subspecies and play an important role in the utilization of indica–japonica heterosis.

Likewise, it is urgent to find out intermediate resources with different degrees of compatibility between the cultivated and wild rice, which will overcome the incompatibility between cultivated and wild rice. In this way, high-quality resources of wild rice can be continuously integrated into cultivated rice.

Furthermore, it is recommended combining transgenic technology and MAS for MDB. The so-called MDB in crop is a novel breeding method with bioinformatics as the platform and genomic and proteomic databases as the basis, which integrates valuable information on crop genetics, physiology and biochemistry, and biological statistics in the crop breeding process. According to the breeding objectives and growth environment of specific crop, MDB designs the optimal scheme and then carries out trials of crop breeding.

MDB mainly involves three steps (Xu and Zhu 2012): (1) to map QTLs for all relevant agronomic traits; (2) to evaluate allelic variation in these QTLs; and (3) to carry out design breeding (Fig. 1). MDB generally has the following requirements: (a) high-density molecular genetic map and high-efficiency molecular marker techniques; (b) sufficient understanding of important genes (QTLs) regarding the location and function; (c) a complete genetic information database established for MDB; (d) a collection of germplasm and intermediate materials applicable for design breeding, including important core germplasm or the backbone parents and their derived RILs with target traits, near-isogenic lines, DH population, chromosome fragment introgression/substitution lines; and (e) improved statistical analysis method and relevant software developed for simulation studies on directed creation of new crop varieties. The above conditions have been met in the case of rice.

The development of rice breeding from conventional genetics to molecular design of new varieties is a general trend, which ensures the breeding of new varieties with improved agronomic traits in terms of the yield, grain quality, select efficiency, disease and pest resistance, and stress tolerance, further contributing to the protection of national food and environmental security.
